# Acute Calculous Cholangitis: Causative Aerobic Bacteria and Antibiotic Susceptibility Patterns from a Retrospective Multicenter Study in Vietnam

**DOI:** 10.3390/life16040599

**Published:** 2026-04-03

**Authors:** Quoc Ai Dang, Thi Thuy Hang Ha, Thi Van Anh Pham

**Affiliations:** 1 Department of General Surgery, E Hospital, Hanoi 100000, Vietnam; dangquocai@hmu.edu.vn; 2 Department of Surgery, Hanoi Medical University, Hanoi 100000, Vietnam; 3 Department of Pharmacy, Hanoi Medical University Hospital, Hanoi 100000, Vietnam; 4 Department of Pharmacology, Hanoi Medical University, Hanoi 100000, Vietnam; phamvananh@hmu.edu.vn

**Keywords:** acute calculous cholangitis, antimicrobial resistance, ESBL, *Escherichia coli*, *Klebsiella* spp., *Enterococcus* spp.

## Abstract

Background: Acute calculous cholangitis is commonly associated with biliary tract infections and is predominantly caused by enteric bacteria. Increasing antimicrobial resistance, particularly among extended-spectrum β-lactamase (ESBL)-producing Gram-negative organisms, has become a major concern in Southeast Asia, including Vietnam. Updated local microbiological data are essential to guide appropriate empirical antibiotic therapy. Methods: This retrospective multicenter study analyzed clinical and microbiological data from patients diagnosed with acute calculous cholangitis. Bacterial culture results were collected from Hanoi Medical University Hospital, Thanh Nhan Hospital, and E Hospital between June 2022 and December 2024. Results: Gram-negative bacteria were predominant (286/366, 78.14%), while Gram-positive bacteria accounted for 80/366 (21.86%). *Escherichia coli* was the most frequently isolated organism (133/366, 36.34%), of which 77/133 (57.89%) were ESBL producing. *Klebsiella* spp. accounted for 60/366 (16.39%), with 17/60 (28.33%) ESBL-producing isolates. *Enterococcus* spp. (n = 80) exhibited high susceptibility to vancomycin (69/80, 86.15%) and complete susceptibility to linezolid (80/80, 100%). Conclusions: This multicenter study highlights evolving pathogen distributions and antimicrobial resistance patterns in acute calculous cholangitis in Vietnam. These findings provide valuable evidence to support the optimization of empirical antibiotic regimens in clinical practice.

## 1. Introduction

Acute calculous cholangitis is an acute inflammatory condition of the biliary tract caused by gallstone obstruction, leading to bile stasis and increased intrabiliary pressure. This obstruction disrupts the normal flushing mechanism of bile and compromises the integrity of the biliary epithelium, facilitating bacterial translocation from the duodenum into the biliary tree. As intrabiliary pressure rises, bacteria and endotoxins may enter the systemic circulation, leading to bacteremia and sepsis. Without timely biliary decompression and appropriate antimicrobial therapy, the infection can rapidly progress to severe systemic inflammatory response, septic shock, and multiorgan failure. Therefore, bacterial invasion plays a central role in determining disease severity and clinical outcomes in acute calculous cholangitis. According to the Tokyo Guidelines 2018, gallstones represent the most common cause of biliary obstruction, accounting for approximately 28–70% of acute cholangitis cases in patients without prior biliary stenting. The diagnosis is based on the presence of systemic inflammation, cholestasis, and imaging findings, classically manifested by Charcot’s triad—fever or chills, jaundice, and right upper quadrant abdominal pain—together with ultrasonographic, computed tomography, or magnetic resonance imaging evidence of biliary stones or ductal dilatation [1]. Gallstone migration from the gallbladder into the common bile duct results in mechanical obstruction and facilitates bacterial colonization of the biliary tree. Without timely treatment, acute calculous cholangitis may progress to severe complications, including sepsis (reported in 25–40% of cases), hepatic abscess, biliary peritonitis, and multiorgan failure, with mortality rates reaching 10–30% in Asia [2,3].

The microbiology of biliary tract infections is heterogeneous but predominantly involves aerobic bacteria of gastrointestinal origin. Gram-negative organisms are most frequently isolated, particularly *Escherichia coli*, followed by *Klebsiella pneumoniae*, *Enterococcus* spp., *Enterobacter* spp., and *Pseudomonas aeruginosa*. These pathogens typically reach the biliary system through ascending infection from the duodenum or via hematogenous spread [4]. Over the past decade, the emergence of extended-spectrum β-lactamase (ESBL)-producing enteric bacteria has posed a major challenge to the management of biliary and other gastrointestinal infections. Global surveillance data indicate that Southeast Asia exhibits one of the highest prevalences of ESBL-producing organisms worldwide, estimated at 7.2% (95% CI, 5.1–9.2%) [5].

Antibiotic therapy is a cornerstone of acute cholangitis management and should be initiated promptly after diagnosis. The increasing prevalence of multidrug-resistant (MDR) organisms in biliary tract infections has further complicated empirical antibiotic selection and may contribute to treatment failure and worse clinical outcomes. Current guidelines recommend administration of empirical antimicrobial agents within 6 h of diagnosis and within 1 h in patients with septic shock [6]. Appropriate empirical antibiotic selection must be guided by local pathogen distributions and antimicrobial susceptibility patterns [7,8], as well as regional resistance trends [9,10]. In Vietnam, however, antibiotic use remains suboptimal [11], and access to newer antimicrobial agents is often limited by healthcare insurance policies. In parallel, rapid bacterial adaptation, including enzyme-mediated antibiotic inactivation, has contributed to a substantial increase in antimicrobial resistance (AMR). Previous studies from Vietnam have reported high resistance rates, particularly among Gram-negative organisms, with resistance to third- and fourth-generation cephalosporins, aminoglycosides, and fluoroquinolones ranging from 40% to 70% [12,13,14].

Therefore, this multicenter study conducted at three tertiary hospitals in Vietnam aimed to characterize the causative aerobic bacterial pathogens and their antimicrobial susceptibility patterns in patients with acute calculous cholangitis, thereby providing evidence to support more effective empirical antibiotic strategies in clinical practice.

## 2. Study Subjects and Methods

### 2.1. Study Subjects

This study included patients diagnosed with acute calculous cholangitis, with or without concomitant acute cholecystitis, in accordance with the diagnostic criteria of the Tokyo Guidelines 2018 [1]. Eligible patients underwent bile sampling for bacterial culture and antimicrobial susceptibility testing at Hanoi Medical University Hospital, Thanh Nhan Hospital, and E Hospital between 1 June 2022, and 31 December 2024 (Table 1).

**Table 1:** Inclusion and Exclusion Criteria of the Study Population.

Category	Criteria
Inclusion Criteria	
Diagnosis	Patients diagnosed with acute calculous cholangitis according to the Tokyo Guidelines 2018 diagnostic criteria.
Disease status	First episode of gallstone-induced acute cholangitis, with or without concomitant acute cholecystitis. Prior antibiotic exposure was not systematically recorded.
Sampling	Bile samples obtained within 24 h of hospital admission during surgical intervention or ultrasound-guided percutaneous biliary or gallbladder drainage.
Microbiology	Bacterial culture and antimicrobial susceptibility testing performed using the VITEK^®^ 2 Compact system (bioMérieux, Craponne, France) and interpreted according to CLSI M100 (2021 edition) [15]. ESBL-producing bacterial strains were identified by the combination disk method and the VITEK automated system.
Study period	Hospitalization between 1 June 2022 and 31 December 2024 at participating centers.
Centers	Hanoi Medical University Hospital, Thanh Nhan Hospital, and E Hospital.
Exclusion Criteria	
Non-bacterial etiology	Bile cultures negative for bacterial growth or indicating non-bacterial causes.
Age	Patients younger than 18 years.
Recurrent disease	Patients with documented recurrent episodes of gallstone-induced cholangitis or cholecystitis during the study period.
Sampling source	Culture specimens obtained from non-biliary sites (e.g., blood, urine, or other body fluids).
Anaerobic bacteria	Isolates of anaerobic bacteria (not included in the present analysis).
Incomplete data	Medical records with missing key clinical or microbiological data.

### 2.2. Research Methods

#### 2.2.1. Study Design

This was a retrospective descriptive cross-sectional study.

#### 2.2.2. Data Collection

Patient data were retrieved from electronic medical record (EMR) systems at the participating hospitals. Cases diagnosed with acute cholangitis were identified based on International Classification of Diseases, 10th Revision (ICD-10) codes between 1 June 2022, and 31 December 2024. First-episode cholangitis was identified based on available electronic medical records; misclassification cannot be completely excluded due to the retrospective design. Patients who underwent bile bacterial culture and identification within 24 h of admission were included. Cases in which culture specimens were obtained from sites other than the biliary tract were excluded.

#### 2.2.3. Study Variables

Collected variables included patient demographics (age and sex), duration of treatment, bacterial culture results, identified bacterial species, presence of extended-spectrum β-lactamase (ESBL)-producing organisms, and antimicrobial susceptibility profiles to commonly used antibiotics.

#### 2.2.4. Statistical Analysis

Data were entered and analyzed using SPSS software (version 20.0; IBM Corp., Armonk, NY, USA). Descriptive statistical methods commonly applied in medical research were used to summarize the study findings.

#### 2.2.5. Ethics Approval

This retrospective study was approved by the Institutional Review Boards of Hanoi Medical University Hospital, Thanh Nhan Hospital, and E Hospital. The requirement for written informed consent was waived because of the retrospective design and the use of anonymized data. The study protocol was approved by the Ethics Committee of E Hospital (Decision No. 2131/QĐ-BVE, dated 6 July 2023).

## 3. Results

### 3.1. Patient Characteristics and Culture Results

From 1 June 2022, to 31 December 2024, a total of 609 medical records of patients diagnosed with acute calculous cholangitis were retrospectively reviewed across three tertiary hospitals: Hanoi Medical University Hospital, Thanh Nhan Hospital, and E Hospital. Among these, 535 patients met the inclusion criteria and underwent bile sampling for bacterial culture and antimicrobial susceptibility testing.

**Figure 1:**
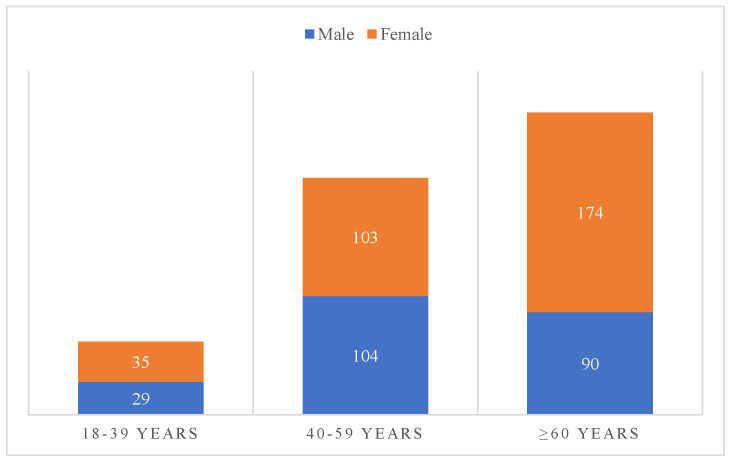
The demographic characteristics of the study.

The mean age of the study population was 60.11 ± 16.18 years (range, 18–95 years). Patients aged ≥60 years accounted for the largest proportion (49.35%, n = 264), followed by those aged 40–59 years (38.69%, n = 207). Female patients were more prevalent than males (58.32% vs. 41.68%). The mean length of hospital stay was 13.41 ± 9.55 days (Figure 1).

**Figure 2:**
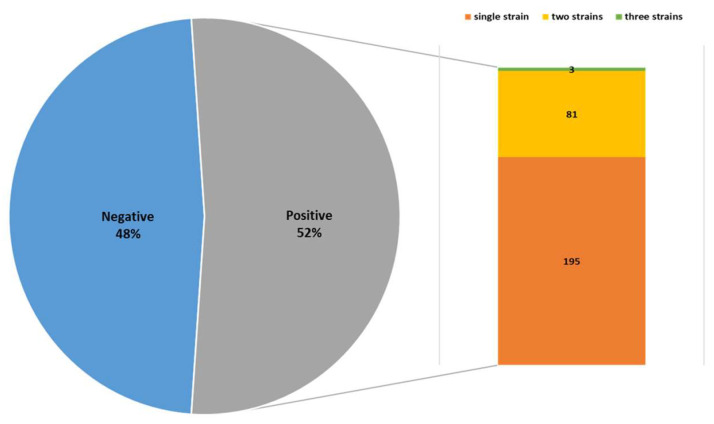
The distribution of bacterial species isolated from bile cultures.

Of the 535 bile samples, 279 (52.15%) yielded positive cultures, whereas 256 samples (47.85%) were culture-negative. Among the positive cultures, 195 samples (69.89%) grew a single bacterial strain, 81 samples (29.03%) yielded two strains, and 3 samples (1.08%) yielded three strains (Figure 2).

### 3.2. Distribution of Isolated Bacterial Strains

A total of 366 bacterial isolates were identified, comprising 16 distinct species. Gram-negative bacteria predominated, accounting for 78.14% of all isolates, whereas Gram-positive bacteria represented 21.86%.

Among Gram-negative organisms, *Escherichia coli* was the most frequently isolated pathogen (36.34%), followed by *Klebsiella* spp. (16.39%), *Pseudomonas* spp. (9.29%), and *Aeromonas hydrophila* (3.01%). Other less common Gram-negative species included *Enterobacter* spp., *Citrobacter* spp., *Morganella morganii, Proteus* spp., and *Acinetobacter* spp. Among Gram-positive bacteria, *Enterococcus* spp. accounted for the majority (19.95%), followed by *Streptococcus* spp. (1.37%) and *Staphylococcus aureus* (0.55%). Detailed distributions are presented in Table 2.

Notably, 33.22% of Gram-negative isolates were extended-spectrum β-lactamase (ESBL) producers. The prevalence of ESBL production was particularly high in *E. coli* 77/133 (57.89%), while *Klebsiella* spp. exhibited ESBL production in 28.33% of isolates.

### 3.3. Antimicrobial Resistance Patterns

**Figure 3:**
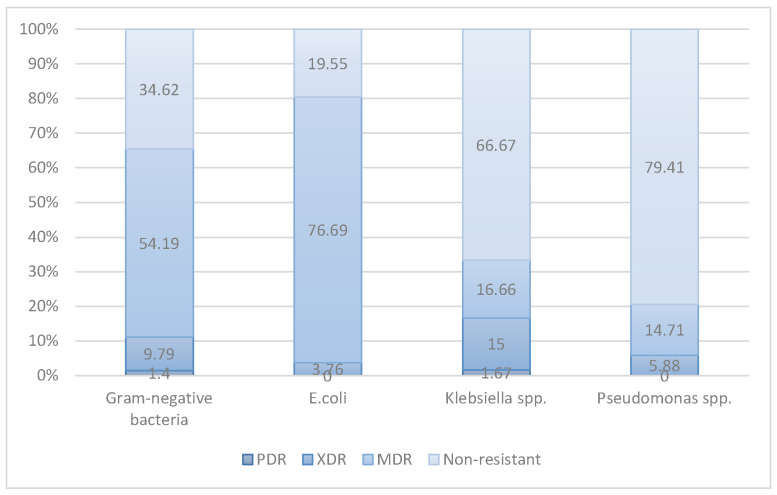
The rate of multidrug-resistant (MDR) for each Gram-negative bacteria strain.

Based on the Antimicrobial resistance of clinical bacterial isolates according to the WHO’s AWaRe and the ECDC-MDR classifications [16], the rate of multidrug-resistant (MDR) Gram-negative bacteria is 54.19% (155/286), among which 9.79% (28/286) are extensively drug-resistant (XDR) and 1.4% (4/286) are pandrug-resistant (PDR) (Figure 3).

The antimicrobial susceptibility profiles of the three most common pathogens—*E. coli*, *Klebsiella* spp., and *Enterococcus* spp.—are summarized in Table 3.

*E. coli* demonstrated high resistance rates to ampicillin/sulbactam (88.37%) and ciprofloxacin (75.57%). In contrast, high susceptibility was observed to carbapenems (>90%), amikacin (99.17%), and fosfomycin (92.42%).

*Klebsiella* spp. showed complete resistance to ampicillin/sulbactam (100%) and substantial resistance to ciprofloxacin (62.07%) and cefotaxime (57.89%). However, full susceptibility to ertapenem (100%) and high susceptibility to amikacin (87.50%) and fosfomycin (73.33%) were observed.

*Enterococcus* spp. exhibited favorable susceptibility profiles, with high sensitivity to linezolid (100%), vancomycin (86.15%), and nitrofurantoin (71.43%), as well as to penicillin/β-lactamase inhibitor combinations (>60%).

## 4. Discussion

Our study showed that the mean age of patients with biliary tract infections was 60.11 ± 16.18 years, with patients aged over 60 years accounting for the largest proportion (49.38%). This finding is consistent with the epidemiological data reported by Tingting Liu et al. (2024) [17]. Female patients predominated, representing 58.32% of cases, while males accounted for 41.68%, a trend that has been consistently reported in multiple international studies and is likely related to the higher prevalence of gallstone disease among women. The average length of hospital stay was 13.41 ± 9.55 days, which was shorter than the 19 days reported by Tingting Liu et al. [17]. Length of hospitalization may be influenced by several factors, including disease severity, patient age, comorbidities, and response to treatment.

Among the 535 bile samples collected within 24 h of hospital admission during diagnostic or surgical procedures, the bacterial culture positivity rate was 52.15%, which is slightly lower than the 55.12% reported by Gu X et al. (2020) [18]. In clinical practice, empirical antibiotic therapy should be initiated as soon as a definitive diagnosis of biliary tract infection is established, and delaying antibiotic administration solely for microbiological sampling is not recommended [19]. Consequently, bile samples are often obtained after antibiotics have already been administered, which may partly explain the relatively modest culture positivity rate observed in this study.

Our findings demonstrated that Gram-negative bacteria were the predominant pathogens, accounting for 78.14% of all isolated strains. This proportion is higher than that reported in a German study, where Gram-negative organisms represented only 43% of isolates [20]. However, our results are consistent with multiple studies from Vietnam and other Asian countries, which have reported Gram-negative bacteria as the dominant causative agents of biliary tract infections, with proportions ranging from 76.8% to 86.8% [21,22]. Among Gram-positive organisms, *Enterococcus* spp. were the most frequently isolated, accounting for 19.95% of total strains. This finding underscores the clinical relevance of *Enterococcus*, particularly *E. faecium* and *E. faecalis*, in biliary tract infections.

Among Gram-negative pathogens, *Escherichia coli* was the most common organism, representing 36.34% of all isolates (133 strains). Notably, the proportion of *Klebsiella* spp. increased from 7.92% to 16.39% in our cohort. This trend is consistent with findings from a 2017 American College of Surgeons study, which reported a gradual decline in *E. coli* as the dominant pathogen and a corresponding increase in other Enterobacteriaceae, including *Enterobacter* spp., as well as non-fermenting Gram-negative bacilli such as Pseudomonas aeruginosa and *Acinetobacter* spp. [19]. In our study, *Klebsiella* spp. accounted for 16.39% of isolates, which is comparable to the distribution reported in the Tokyo Guidelines 2018 [4]. Among these, *K. pneumoniae* was the most common species (75%), followed by *K. aerogenes* (13.33%) and *K. oxytoca* (11.67%).

Antimicrobial resistance among Gram-negative bacteria causing acute biliary tract infections, particularly extended-spectrum beta-lactamase (ESBL) production, has emerged as a major global concern. A retrospective study in Germany by Reuken et al. reported that ESBL-producing organisms accounted for 54% of positive bile cultures [23]. In South Korea, ESBL-producing *E. coli* has been identified as the leading cause of acute cholangitis, including community-acquired cases, with a reported prevalence of 30.4% [24]. In the present study, 57.89% of *E. coli* isolates and 28.33% of *Klebsiella* spp. isolates were ESBL producers. These rates were lower than those reported in a multicenter study from China, where ESBL production was detected in 62.55% of *E. coli* strains and 88.68% of *K. pneumoniae* strains [25]. Due to the lack of standardized disease severity grading according to the Tokyo Guidelines 2018 across participating centers, we were unable to analyze resistance patterns or ESBL prevalence in relation to cholangitis severity or clinical outcomes. Consequently, whether resistant pathogens are associated with more severe disease courses could not be assessed. Furthermore, the heterogeneity of microbiological testing methods between centers precluded inferential analyses and subgroup comparisons (e.g., ESBL vs. non-ESBL isolates, inter-hospital differences). As a result, the study was limited to descriptive statistics, which may constrain the identification of predictors associated with resistance patterns. These limitations highlight the need for future multicenter prospective studies with standardized protocols to enable robust comparative analyses.

A notable point of the present study is the lack of anaerobic bacterial identification, as routine anaerobic bile culture is not commonly performed in Vietnam. Given evidence from the Tokyo Guidelines 2018 indicating that anaerobes contribute to 4–20% of biliary tract infections [4], the findings reported here should be interpreted with caution when selecting regimens targeting anaerobic pathogens.

The rate of multidrug-resistant (MDR) Gram-negative bacteria in biliary tract infections was 54.19%, with XDR and PDR rates of 9.79% and 1.4%, respectively. Compared to a 2025 international multicenter study (with MDR rates ranging from 7.21% to 21.16%) [26], the MDR rate in this study is significantly higher, particularly for *E. coli* (76.69% vs. 11.34–30.70%). *Klebsiella* spp. exhibited the highest XDR rate (15%) and was the only strain with PDR (1.67%), reflecting the risk from carbapenemase-carrying lineages. In contrast, Pseudomonas spp. maintained a high susceptibility rate (79.41%), consistent with the general trend in Europe [27,28]. These findings emphasize the need for continuous antimicrobial surveillance and the adjustment of empirical regimens based on local microbiological data.

Analysis of antimicrobial susceptibility patterns showed that *E. coli* remained highly sensitive to amikacin (99.17%), nitrofurantoin (100%), and fosfomycin (92.42%). Although susceptibility data for nitrofurantoin and fosfomycin are presented, these agents have limited biliary penetration and are not recommended for the treatment of biliary tract infections. Their inclusion reflects microbiological susceptibility testing results rather than clinical treatment recommendation. Susceptibility to carbapenems was also high, ranging from 92.42% to 98.25%. These findings are consistent with those reported by Zhao C. et al. (2022) in China, where *E. coli* demonstrated sensitivity rates above 90% to ertapenem, imipenem, tigecycline, amikacin, and piperacillin/tazobactam [21]. In contrast, *E. coli* exhibited high resistance to ampicillin/sulbactam (88.37%), ciprofloxacin (75.57%), and cefotaxime (71.32%). Given the high resistance rates of *E. coli* to ciprofloxacin observed in this study, fluoroquinolones may no longer be appropriate for empirical therapy of acute calculous cholangitis in the local setting. Empirical regimens should prioritize agents with preserved activity against prevalent Gram-negative pathogens, while being adapted to local antimicrobial stewardship policies and updated resistance surveillance.

For *Klebsiella* spp., susceptibility remained high to ertapenem (100%), amikacin (87.50%), and fosfomycin (73.33%). However, reduced carbapenem susceptibility in *Klebsiella* spp.: imipenem/cilastatin (65.52%) and meropenem (71.67%). *Klebsiella* spp. intrinsically resistant to ampicillin/sulbactam. High resistance rates were also noted for ciprofloxacin (62.07%), cefotaxime (57.89%), and trimethoprim/sulfamethoxazole (65.33%). These findings contrast with data from Zhao C. et al. (2022), who reported full susceptibility of *K. pneumoniae* to carbapenems and amikacin, with lower resistance rates to cephalosporins and sulfamethoxazole [21]. The contrasting resistance patterns compared with previous studies may be attributed to differences in local antibiotic usage, selective pressure, patient populations, and institutional antimicrobial stewardship practices.

Gram-positive bacteria, particularly *Enterococcus* spp., are increasingly recognized as clinically important pathogens in biliary tract infections, as reported in several previous studies [20,29]. This raises concerns regarding the adequacy of commonly used empirical monotherapy regimens, such as third-generation cephalosporins or beta-lactam/beta-lactamase inhibitor combinations, due to the intrinsic resistance of *Enterococcus* to cephalosporins. In our study, these combinations demonstrated susceptibility rates exceeding 60% against *Enterococcus* spp. In contrast, agents specifically targeting Gram-positive bacteria, such as linezolid (100%) and vancomycin (86.15%), showed superior activity, consistent with earlier reports [30].

Overall, resistance rates among *Enterococcus* spp. isolates in our cohort were relatively low, with most resistance rates below 50%. The highest resistance was observed for fluoroquinolones, including ciprofloxacin (53.33%) and levofloxacin (47.83%), as well as tetracycline (52.17%) and penicillin G (50.70%).

This study provides updated microbiological and antimicrobial susceptibility data to inform local empirical antibiotic selection; however, as a retrospective descriptive study without outcome correlation, it cannot determine the clinical superiority of specific regimens. Prospective studies linking empirical therapy to clinical outcomes such as mortality, ICU admission, and treatment failure are needed.

## 5. Conclusions

This study demonstrates that Gram-negative bacteria, particularly *Escherichia coli* and *Klebsiella* spp., are the predominant causative agents of acute calculous cholangitis. In addition, *Enterococcus* spp. represent an important Gram-positive pathogen with an increasing prevalence. High rate of multidrug-resistant (MDR), especially among ESBL-producing Gram-negative strains, remains a major clinical concern. Although *E. coli* and *Klebsiella* spp. retain high susceptibility to several antibiotics, resistance rates to commonly used agents such as ampicillin/sulbactam and ciprofloxacin are notably high. While *Enterococcus* spp. generally exhibit lower resistance rates, their presence still warrants careful consideration in empirical and targeted antibiotic selection. These findings support local antimicrobial stewardship efforts by providing contemporary susceptibility patterns to guide empirical antibiotic selection, while clinical outcome-based validation is warranted in future studies.

## Figures and Tables

**Table 2:** Proportion of Isolated Bacterial Species.

Bacterial Strain	Quantity	Frequency (%)
**Gram-Negative Bacteria**		
*Escherichia coli*	133	36.34
*Klebsiella* spp.	60	16.39
*Pseudomonas* spp.	34	9.29
*Aeromonas hydrophila*	11	3.01
*Enterobacter* spp.	10	2.73
*Citrobacter*	10	2.73
*Morganella morganii*	9	2.46
*Proteus* spp.	7	1.91
*Acinetobacter* spp.	6	1.64
*Stenotrophomonas maltophilia*	3	0.82
*Serratia fonticola*	1	0.27
*Burkholderia cepacia*	1	0.27
*Edwardsiella hoshinae*	1	0.27
**Gram-positive Bacteria**		
*Enterococcus* spp.	73	19.95
*Streptococcus* spp.	5	1.37
*Staphylococcus aureus*	2	0.55
**Total**	366	100

**Table 3:** Resistance, Intermediate, and Sensitivity Rates of *E. coli, Klebsiella* spp., and *Enterococcus* spp.

Antibiotic	*Escherichia coli*				*Klebsiella* spp.				*Enterococcus* spp.			
	(n)	R (%)	I (%)	S (%)	(n)	R (%)	I (%)	S (%)	(n)	R (%)	I (%)	S (%)
Fosfomycin	66	7.58		92.42	30	23.33	3.33	73.33				
Penicillin G									71	50.70	0	49.30
Ampicillin/Sulbactam	129	88.37	0.78	10.85	13	100			71	36.62	2.82	60.56
Amoxicillin/clavulanic	89	34.83	20.22	44.94					46	39.13	0	60.87
Piperacillin/tazobactam	91	23.08	6.59	70.33	41	34.15	19.51	46.34	48	39.58	0	60.42
Cefotaxime	129	71.32	0.78	27.91	57	57.89	3.51	38.60				
Cefepime	132	30.30	9.85	59.85	59	38.98	3.39	57.63				
Ertapenem	114	0.88	0.88	98.25	40			100				
Imipenem/cilastatin	132	7.58	0	92.42	58	29.31	5.17	65.52				
Meropenem	132	7.58	0	92.42	60	28.33	0	71.67				
Amikacin	121	0	0.83	99.17	56	8.93	3.57	87.50				
Gentamicin	133	33.08	0	66.92	59	22.03	3.39	74.58	23	39.13	0	60.87
Ciprofloxacin	131	75.57	6.11	18.32	58	62.07	5.17	32.76	45	53.33	15.56	31.11
Levofloxacin									46	47.83	15.22	36.96
Linezolid									58	0	0	100
Vancomycin									65	12.31	1.54	86.15
Tetracyclin									46	52.17	0	47.83
Nitrofurantoin	6	0	0	100					21	19.05	9.52	71.43
Trimethoprim/ sulfamethoxazole	132	70.45	0	29.55	60	53.33	0	46.67				

R: Resistant; I: Intermediate; S: Sensitive. (Not all isolates were tested against all antibiotics due to reagent availability and institutional protocols.)

## Data Availability

The data presented in this study are available from the corresponding author upon reasonable request. The data are not publicly available due to ethical restrictions and the protection of patient privacy.
